# Engineering Functional Vasculature in Decellularized Lungs Depends on Comprehensive Endothelial Cell Tropism

**DOI:** 10.3389/fbioe.2021.727869

**Published:** 2021-08-16

**Authors:** Ifeolu Akinnola, Daniel R. Rossi, Carolyn Meyer, Ashley Lindsey, Douglas R. Haase, Samuel Fogas, Michael J. Ehrhardt, Rachel E. Blue, Andrew. P. Price, Max Johnson, Diego F. Alvarez, Doris A. Taylor, Angela Panoskaltsis-Mortari

**Affiliations:** ^1^ MSTP, University of Minnesota Medical School, Minneapolis, MN, United States; ^2^ Pediatric Blood and Marrow Transplantation and Cell Therapy, University of Minnesota, Minneapolis, MN, United States; ^3^ Internal Medicine and Center for Lung Biology, College of Medicine, University of South Alabama, Mobile, AL, United States; ^4^ University of Minnesota Medical School, Minneapolis, MN, United States; ^5^ RegenMedix, Houston, TX, United States; ^6^ Pulmonary, Allergy, Critical Care and Sleep Medicine, University of Minnesota, Minneapolis, MN, United States

**Keywords:** endothelial progenitors, decellularization, extracellular matrix, tissue engineering, bioscaffold recellularization

## Abstract

Tissue engineering using decellularized whole lungs as matrix scaffolds began as a promise for creating autologous transplantable lungs for patients with end-stage lung disease and can also be used to study strategies for lung regeneration. Vascularization remains a critical component for all solid organ bioengineering, yet there has been limited success in generating functional re-endothelialization of most pulmonary vascular segments. We evaluated recellularization of the blood vessel conduits of acellular mouse scaffolds with highly proliferating, rat pulmonary microvascular endothelial progenitor cells (RMEPCs), pulmonary arterial endothelial cells (PAECs) or microvascular endothelial cells (MVECs). After 8 days of pulsatile perfusion, histological analysis showed that PAECs and MVECs possessed selective tropism for larger vessels or microvasculature, respectively. In contrast, RMEPCs lacked site preference and repopulated all vascular segments. RMEPC-derived endothelium exhibited thrombomodulin activity, expression of junctional genes, ability to synthesize endothelial signaling molecules, and formation of a restrictive barrier. The RMEPC phenotype described here could be useful for identifying endothelial progenitors suitable for efficient vascular organ and tissue engineering, regeneration and repair.

## Introduction

The treatment of end-stage lung diseases requires lung transplantation, yet there is a severe shortage of available lungs for transplant. Roughly 20% of patients on lung transplant lists are removed before receiving a new lung due to death or becoming too ill to undergo the procedure (Ahya and Diamond, 2019). Tissue engineering using decellularized whole lungs as scaffolding might provide an opportunity to generate functional lungs for autologous transplant in patients for whom there is no other therapeutic alternative. Despite some advancements in lung tissue engineering (Petersen et al., 2010; Song et al., 2011; Calle et al., 2014; Ren et al., 2015; Balestrini et al., 2016), to date, the establishment of a functioning vasculature with low thrombogenicity and adequate barrier function has proven to be difficult, resulting in lung failure. Although synthetic vascular grafts are utilized clinically for simple vessels (Deutsch et al., 2009) current synthetic materials can mimic neither the anatomical placement of cells interacting at the air-liquid interface, the native mechanical properties of the lung, nor the complex organ compositional and micro-environmental cues needed for geospatial branching. Perfusion-based decellularization provides an alternative approach that preserves the lung’s native architecture and extracellular matrix composition, overcoming these challenges (Price et al., 2010; Daly et al., 2012). Decellularized lungs provide the ideal biomimetic cues for appropriate cell localization, differentiation, and function. Such three-dimensional niches are conducive to physiological cell-matrix and cell-cell crosstalk, potentially enhancing the functionality and stability of engineered, transplantable organs.

Whole complex solid organ regeneration, while still in the early stages, has rapidly matured since the initial demonstration for the heart (Ott et al., 2008; Gilpin et al., 2016). Bioengineered lungs implanted in rats showed evidence of successful gas exchange for up to 7 days (Song et al., 2011), and a similar method is being developed using a nonhuman primate model (Bonvillain et al., 2012). Bioengineered lungs have also been successfully implanted into large, non-primate mammals lasting up to 2 months, without acute rejection, where the implanted lung displayed continued growth and restoration of native microbiota (Nichols et al., 2018). While the longevity of a lung transplant depends on many different factors, the functionality of engineered grafts has faltered primarily from their susceptibility to thrombus formation and barrier leakiness (Petersen et al., 2010; Kurobe et al., 2012; Balestrini et al., 2016; Liu et al., 2018). In native tissues, the endothelium regulates hemostasis and thrombosis, forming a functional barrier that allows for selective permeability (Feletou and Vanhoutte, 2006; Aird, 2007). The primary goal of this study was to determine the endothelial cell specification necessary to generate functional, patent pulmonary vasculature using acellular whole lung matrices in bioreactor conditions. We show the identification of a pulmonary endothelial cell population from a single source that can overcome selective tropism, recellularize all vascular areas of an acellular lung scaffold, and provide a functional barrier.

## Methods

Animals: Male B10.BR mice were bred in-house at the University of Minnesota Research Animal Resources facility and used at 8–12 weeks of age. Sprague Dawley rats were purchased from Charles River Laboratory, housed at University of South Alabama and used at 4 months of age. All experiments were approved by the Institutional Animal Care and Use Committees of the respective institutions.

Perfusion based decellularization of whole mouse lungs: Mouse lungs were decellularized as previously published (Price et al., 2010). Lungs were excised *en bloc* with the trachea and heart, and were perfused with sequential solutions of deionized water, 0.1% Triton X-100, 1% 3α,12α-dihydroxy-5β-cholanic acid sodium salt (Na deoxycholate), 1 M NaCl, then 30 mg/ml porcine pancreatic DNase in 1.3 mM MgSO_4_ and 2 mM CaCl_2_, followed by a final rinse with sterile, deionized water. Cell removal was verified via 4′,6-diamidino-2-phenylindole (DAPI) immunofluorescence (Vector Labs) confocal microscopy (Olympus FV500) on cryosections of representative lungs processed at the same time for quality control purposes, as well as DNA quantitation using the PicoGreen Assay (Price et al., 2015). Decellularized scaffolds were maintained at 4°C in PBS containing pen/strep to maintain sterility.

Isolation and culture of rat endothelial cells: PAECs, MVECs, and RMEPCs were isolated from Sprague-Dawley rats and cultured as previously described (King et al., 2004; Alvarez et al., 2008). For PAECs, the main pulmonary artery was dissected from the root to a second vessel generation and under sterile conditions placed in a 60 mm dish containing ice-cold DMEM (GIBCO). The vessels were inverted, the intima was scraped, and the collected cells were strained with a 20 µm filter (BD Biosciences). Harvested cells were transferred to a T75 flask, supplemented with DMEM enriched with 20% FBS (Hyclone) and 100 U/ml penicillin-100 μg/ml streptomycin (GIBCO) and were incubated at 37°C with 5% CO_2_-21% O_2_. For MVECs, the distal lung parenchyma was sliced, and the subjacent tissue was carefully dissected and placed in a 60-mm dish containing cold DMEM (4°C). Tissue was digested with type II collagenase (Worthington), rinsed with DMEM, transferred to a T75 flask, and incubated as described for PAECs. Cell culture medium was replaced with DMEM + 10% FBS and 1% penicillin/streptomycin and passages 5–7 were used for consecutive experiments. Cells were selected for homogeneity based on morphological appearance and tested by flow cytometry as described below. For RMEPCs, cells were selected after subjecting MVECs to a single-cell clonogenic assay as described (Alvarez et al., 2008). All cells were screened for *mycoplasma* (Lonza #LT07) and found to be negative.

Flow cytometry characterization of rat endothelial cells: PAECs, MVECs, RMEPCs were characterized by flow cytometry using a FACSCalibur (BD Biosciences) and analyzed with Cyflogic software. Cells were phenotyped using antibodies to rat CD31 (Abcam #ab28364), CD34 (BD Biosciences #341071), CD62P (Takara Bio #M062), CD104 (Millipore #AB 1922), CD146 (R&D Systems #FAB3250P), eNOS (Santa Cruz #sc654), VE-Cadherin (Santa Cruz #sc9989), VEGF (Santa Cruz #sc507), vWF (Affinity Biologicals #SARTW), and lectins from *Griffonia simplicifolia I and II* (GSI and GSII, EY Laboratories #F-2401-B-1 and #R2402-2), and *Helix pomatia* (HP, EY Laboratories #R3601-1 and F3601-1).

Gene expression: mRNA from recellularized lungs and cells grown in tissue culture flasks was analyzed using the RT2 Profiler PCR Array for Rat Tight Junctions (Qiagen, Valencia, CA). cDNA was generated using the RT2 First Strand Kit (Qiagen) following the product insert. Quantitative real-time PCR was performed on an ABI 7500 Real Time PCR System using the RT2 SYBR Green ROX qPCR MasterMix (Qiagen). Results were entered into the Ingenuity pathway analysis program (Ingenuity Systems, Inc.). Relative gene expression analyses of LDL-receptor, scavenger receptors and integrins was done by isolating total RNA from cells with TRIzol (Invitrogen, Carlsbad, CA), generating cDNA using the Superscript III First Strand RT PCR kit (Life Technologies), and qRT-PCR done using Taqman Universal PCR Mastermix (Life Technologies). For analysis, samples were only used in comparison if they met our cutoff criteria (i.e. CT values ≤ 35 and dCT Std Err ≤0.25). The probes used for these analyses were ordered from Life Technologies (Grand Island, NY) and listed in Supplementary Table S5. For analysis of LDL-receptor and scavenger gene expression, samples from each cell line underwent serum starvation overnight prior to obtaining total RNA.

*In vitro* tube formation assay: Tube formation assays were performed as previously published (Chung-Welch et al., 1989). PAECs, MVECs, and RMEPCs were seeded at 4 × 10^4^ cells per well, in triplicate, of a 24-well plate coated with 100 μl/cm^2^ BD Matrigel (BD Biosciences) and incubated at 37°C, 5% CO_2_. Cultures were imaged with a phase-contrast microscope (Leica) 2, 4, 8, and 24 h post-seeding.

Assessment of endothelial function: Dil-Ac LDL (1,1′-dioctacecyl-1,3,3,3′3-tetramethyl-indocarbocyanine perchlorate acetylated low density lipoprotein) uptake in PAECs, MVECs, and RMEPCs was measured in confluent cultures using previously published methods (Chang et al., 2009). Cultured cells were incubated with 10 μg/ml Dil-Ac LDL (ThermoFisher) for 4 h before analyzing for fluorescence at 550 nm (Leica). For reseeded lung scaffolds, 10 μg/ml Dil-Ac LDL was added to the bioreactor media 7 days after seeding to assess uptake. To prepare for histological analysis, lung constructs were inflated with PBS:OCT (1:3 ratio), embedded in OCT, frozen in liquid nitrogen and stored at -80°C. Cryosections (7 µm) were air dried, fixed with methanol (4°C) for 10 min and air dried for 30 min. Slides were then rinsed twice with PBS for 3 min, then with PBS-Tween 20 (0.2 M PBS, 0.05% Tween 20, pH 7.4) twice for 2 min before coverslipping with VectaShield mounting medium (Vector Labs) containing nucleic acid stain 4′,6-diamidino-2-phenylindole (DAPI) and imaged using a confocal microscope (Olympus FV500).

Bioreactor assembly: T-25 flasks were modified in sterile conditions. Holes were created through the vented filter cap and on the bottom of one wall just large enough to allow passage of 2 mm outer diameter Puri-Flex tubing (Masterflex) through both holes. This allowed for continuous circulation of liquid out from the bottom of the bioreactor, through an adjustable Masterflex pump, and back in through the cap, terminating with a 20 gauge x 1 ½″ disposable murine gavage needle (Cadence Science) serving as a cannula for the heart/lung bloc. A hole was created in the right ventricular wall to allow insertion of the rubberized gavage needle tip to access the pulmonary trunk (main pulmonary artery).

**FIGURE 1:**
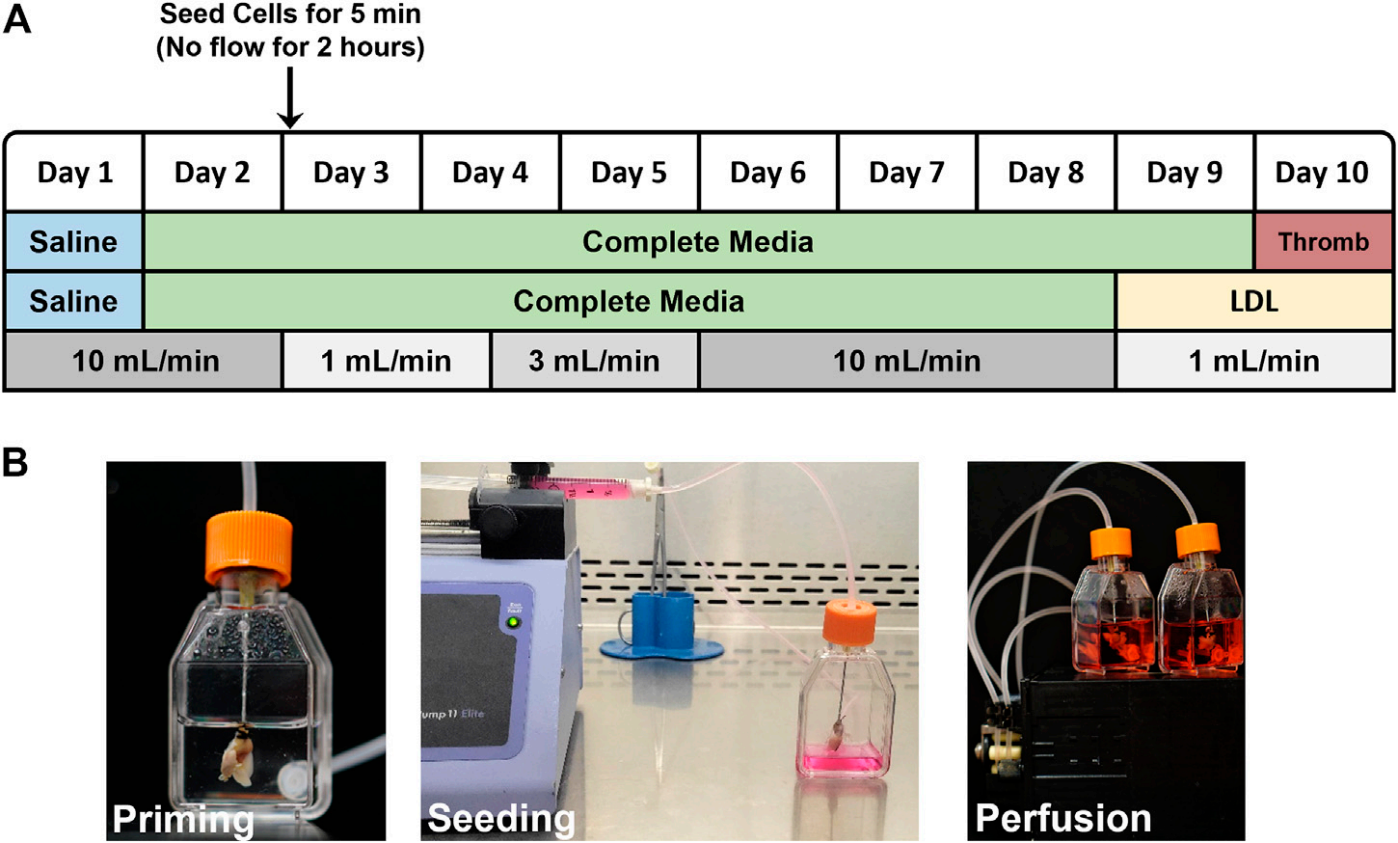
**(A)** Flow chart outlining the reseeding protocol. LDL-uptake and Thrombogenicity assays were conducted on day 9 and 10, respectively. **(B)** Photographs showing assembled, modified tissue culture flask bioreactor with attached acellular mouse lung scaffold during priming with saline **(left)**, injector pump seeding ECs into acellular scaffold via right ventricle **(middle)**, and reseeded lung scaffolds with 25 ml of media in a closed loop system, attached to pulsatile pump **(right)**.

Reseeding decellularized lungs with endothelial cells: The bioreactor set up, cell seeding protocol and assay endpoints are shown in Figure 1A. Decellularized lungs were perfused in a humidified bioreactor at 37°C/5%CO_2_/21%O_2_ with 25 ml PBS for 1 day (24 h), then with 25 ml of DMEM containing 10% FBS and 1% penicillin-streptomycin (complete media) for 1 day (24 h) prior to reseeding. Endothelial cells were washed with PBS and lifted from theirz tissue culture dish via 0.05% trypsin/EDTA, centrifuged for 5 min at ×2,000 g, and resuspended at 1 × 10^7^ cells/ml. The cell suspension was filtered using a 40 µm pore mesh and loaded in a 3 ml luer lock syringe, for a total of 3 × 10^7^ cells injected into each pair of decellularized lungs. The 20 gauge x 1 ½″ disposable gavage needle cannulating the decellularized heart/lung bloc was then attached to the syringe and placed in an automated syringe pump set at 0.6 ml/min. During the 5 min cell infusion, a 15 ml conical tube was placed under the lungs to collect flow for a final cell count and viability analysis. 25ml of fresh complete media was then added to each flask. After cell infusion, the gavage needle was removed from the luer lock syringe and attached to the bioreactor tubing. Air bubbles were avoided by priming the tubing before connections were made. The reseeded lungs were incubated in a humidified chamber at 37°C/5%CO_2_/21% O_2_ for 2 h prior to beginning vascular perfusion. Flow rates began at 1 ml/min on Day 3 (for 36 h), increased to 3 ml/min on Day 4 (for 36 h), increased again to 10 ml/min on Day 6 (for 72 h), and then slowed to 1 ml/min on Day 9 prior to the start of the acetylated LDL uptake or thrombogenicity assays. Media was replaced 24 h after cell seeding and then every 48 h thereafter. The lungs were then removed from the bioreactor to be frozen. Frozen lungs were rinsed with PBS, infused with PBS:OCT (1:3 ratio), embedded in OCT, flash frozen in liquid N_2_ and stored at −80°C.

H&E and Immunofluorescence staining of reseeded lungs: Frozen lung cryosections (7 µm) were prepared as described above. Sections were blocked with 10% normal horse serum for 30 min at room temp and incubated in the dark overnight at 4°C with 1 μg/ml rabbit-anti- VEGF (Santa Cruz #sc507) and sheep-anti-vWF (Affinity Biologicals #SARTW) primary antibodies. Sections were rinsed with PBS-Tween 20 before incubating 30 min at room temp with 1:1,000 Goat Anti-Rabbit IgG TRITC (Jackson #111-025-003) and Donkey Anti-Sheep IgG FITC (R&D Systems #F012F) secondary antibodies, then rinsed 3 times with PBS-Tween 20 for 2 min before coverslipping with VectaShield mounting medium (Vector Labs) containing DAPI. Absence of mouse cells was confirmed by negative PCR for murine GAPDH as described (Price et al., 2010).

Thrombomodulin activity assay: Measuring function of thrombomodulin (CD141) on endothelial cells was adapted from previously published studies (Calnek and Grinnell, 1998; Ibrahim and Ramamurthi, 2008; Robertson et al., 2014). Positive control lungs were excised from euthanized (sodium pentobarbital, 50 mg/kg ip) adult male B10.BR mice with the trachea and heart attached; after exsanguination via the right ventricle, the heart-lung bloc was perfused with PBS. After 7 days in bioreactor (Day 10), all lung (controls and reseeded) constructs were perfused for 15 min at 3 ml/min with 25 ml phenol red-free DMEM/F12. They were then perfused for 45 min at 1 ml/min with 4 ml phenol red-free DMEM/F12 plus human α-thrombin (0.1 U/ml) and human protein C (12 μg/ml). In triplicate, 100 µl was transferred to a 96-well plate and incubated for 5 min at 37°C with excess hirudin (6 U/ml) to inactivate the α-thrombin. S-2366 (Chromogenix) was added for a final concentration of 0.75 mM and incubated at room temperature for 5min before measuring absorbance at λ = 410 nm and λ = 490 nm (BIORAD Model 550). The relative absorbance was calculated by subtracting the mean acellular absorbance (λ = 410 nm from λ = 490 nm) from each sample.

Vascular permeability assay: To assess vascular barrier function in recellularized lungs, Evans blue (EB)-tagged bovine serum albumin (BSA) was perfused through the vasculature and measured using a near-infrared spectrophotometry method adapted from previously published studies (Lowe et al., 2010; von Drygalski et al., 2012). Decellularized and recellularized, and exsanguinated control mouse lung/heart blocs were weighed and perfused for 5 min with phenol red-free DMEM, then for 15 min with 0.4% EB-tagged BSA (EB-BSA) in phenol red-free DMEM, followed by a 5 min perfusion with 4% BSA in PBS before immersing and gently shaking in PBS as a final rinse. The hearts were then removed and weighed to obtain accurate lung weights for data normalization. EB-BSA content of each lung was then analyzed immediately using a Li-COR Odyssey scanner reading at 700 nm at a resolution of 169 µm and “Intensity” = 2. The lungs were scanned at three distinct focal planes (2, 3, and 4 mm) and the average of the three planes was then normalized to lung weight for a final measure of EB-BSA permeability.

In addition to evaluating EB-BSA permeability of recellularized lungs, isogravimetric and lung perfusion methods were used to recorded absolute change in weight of RMEPC-recellularized, decellularized and native mouse lungs. Gavage needles were inserted though the right ventricles in order to use the heart as conduit to the pulmonary vasculature. The trachea was then attached to an isometric force transducer (Harvard Apparatus) with a suture. 2 mm OD Masterflex tubing submerged in a 50 ml conical filled with PBS was primed and connected to the gavage needle while a P75 Hugo Sachs Elektronik pressure transducer (Harvard Apparatus) was added inline. A peristaltic pump was turned on to begin perfusion of the pulmonary vasculature with PBS. Every 5 min, measurements from the pressure and force transducers were taken, followed by an increase in flow rate. This process continued until maximum flow rate from the pump was achieved. Data Acquisition ActiveX™ (DTx-EZ, Quick DataAcq) was used to record measurements from the pressure transducer. Results were recorded into Microsoft Excel, where average slope of each sample (∆V_Force_/∆V_Pressure_) and the average for each group was calculated.

Cytokine assays: Supernatants from endothelial cell cultures and effluent collected from decellularized and recellularized lungs (48 h collections) were assessed for levels of cytokines and soluble mediators that have been shown to play roles in angiogenesis, vascular integrity and permeability. Samples were evaluated for CXCL1, TIMP1, PAI1, and VEGF via the Luminex platform (Austin, TX) using rat-specific bead sets from Millipore (Billerica, MA) and data analyzed with BioPlex software (BioRad, Hercules, CA).

### Statistics

Statistical significance comparisons between control, decellularized, and recellularized lungs were analyzed by using one-way ANOVA using Tukey as a post-hoc test (R x64 2.15.0 statistical software). *p* < 0.05 was considered significant.

## Results

### Characterization of Pulmonary Endothelial Cells

**FIGURE 2:**
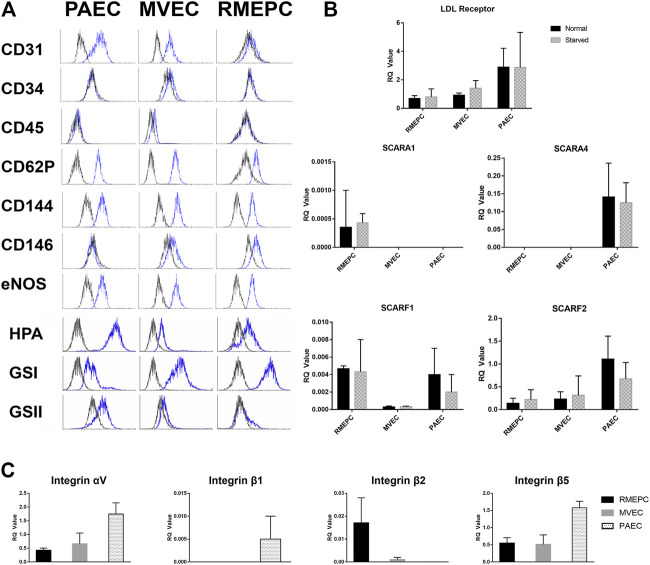
PAEC, MVEC, and RMEPC express representative endothelial cell associated markers and lectin binding properties as assessed via flow cytometry **(A)**. In addition, gene expression of LDL-receptor, selected scavenger receptors, and selected integrin chains were assessed via qPCR **(B,C)**. For qPCR data, *n* = 3 for each sample/condition.

All cultured rat pulmonary endothelial cells used in these studies were capable of *in vitro* tube formation in Matrigel (Supplementary Figure S1A) as previously demonstrated (Alvarez et al., 2008). Functionality of the cultured rat endothelial cells was confirmed *in vitro* by demonstrating uptake of Dil-Ac LDL (Supplementary Figure S1B). In addition, PAECs, MVECs, and RMEPCs were assessed by flow cytometry to confirm the phenotypes perfused into the decellularized lungs. RMEPCs were negative for CD31 (PECAM-1), whereas PAECs and MVECs stained positively (Figure 2). RMEPCs expressed less CD62P (P-selectin), and more CD146 (MUC18) when compared to PAECs and MVECs. Consistent with their endothelial phenotype, all cells displayed high levels of CD144 (VE-Cadherin) and eNOS, and lacked expression of CD34 (mucosialin) and CD45 (leukocyte common antigen). RMEPCs and MVECs were differentiated from their PAEC counterpart by their higher binding to lectins GSI, with low or negative binding to GSII and HPA, consistent with our previous report (Alvarez et al., 2008).

Gene expression of LDL-receptor, multiple scavenger receptors, as well as multiple integrin chains were assessed within PAECs, MVECs, and RMEPCs via qPCR within 2D cell culture. Fold change of genes for LDL-receptor and selected scavenger receptors (i.e. Scara1, Scara4, Scarf1 and Scarf2) were measured in both normal and serum starved conditions. Within both normal and serum starved conditions, all three pulmonary endothelial cell types had measurable expression of LDL-receptor, scavenger receptor class F, member 1 (Scarf1) and class F, member 2 (Scarf2). RMEPCs were the only cell type with measurable expression of Scara1 while PAECs were the only cell type with measurable expression of Scara4. For expression of selected integrin chains (i.e. αv, β1, β2, and β5), PAECs displayed the highest relative expression of αv, β1and β5. RMEPCs had the highest relative expression of β2 and virtually no expression of β1. Reported RQ values were normalized to fresh rat lung, which is comprised of multiple cell types, thus accounting for seemingly lower RQ values amongst some of our target genes.

From the assessment of endothelial markers and gene expression, we have established the overall phenotype of our RMEPCs as CD31^−^, CD45^−^, CD144^+^, eNOS^+^, CD62P^lo^ cells with strong and weak binding to GSI and HPA, respectively. In addition, they have relatively higher gene expression of Scara1 and Itg-β2, compared to PAECs and MVECs.

### Selective populations of endothelial cells and progenitor cells are necessary for segment-specific revascularization of decellularized lungs

**FIGURE 3:**
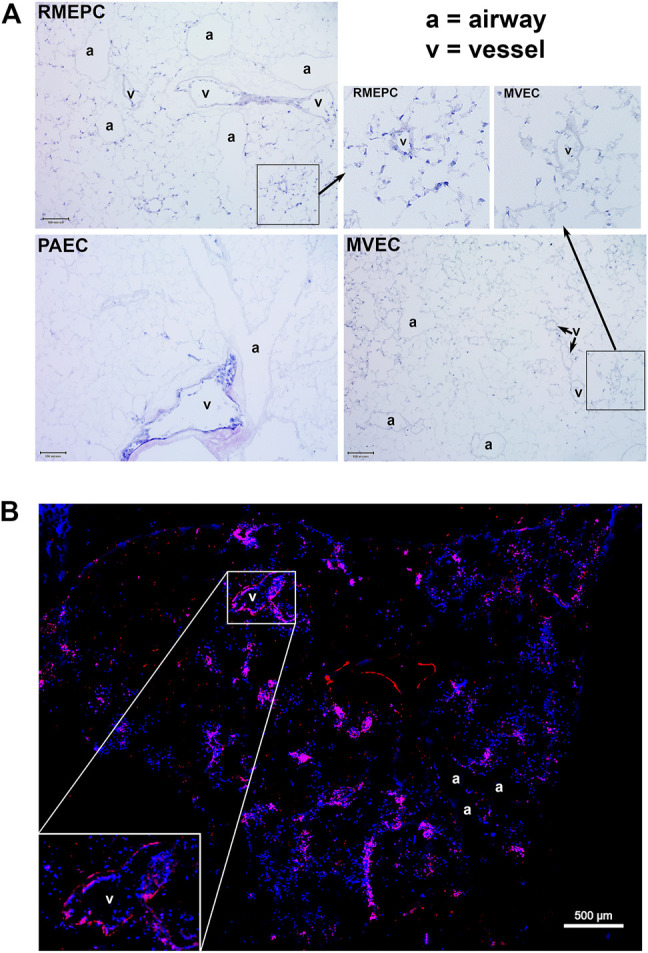
**(A)** MVEC and PAEC show selective tropism to their *in vivo* point of origin whereas RMEPC exhibit pan-tropism to *ex vivo* decellularized ECM vascular conduits. Representative H&E-stained cryosections (5 um) of decellularized whole lungs re-seeded with progenitor enriched microvascular cells (RMEPC), pulmonary arterial endothelial cells (PAEC), and microvascular (MVEC). All images are at ×100 magnification (×10 objective). Scale bar indicates 100 µm. Arrows indicate zoomed-in areas comparing engraftment of RMPEC to microvasculature and larger vessel, whereas MVEC engraftment is only in microvasculature. **(B)** Immunofluorescent image of a decellularized mouse lung lobe seeded with RMEPCs. Cryosection was stained for VE-Cadherin (red). Multiple images taken at ×40 objective magnification and stitched together. DAPI used to label cell nuclei (blue).

PAECs, MVECs and RMEPCs were infused into the lung vasculature via the pulmonary artery trunk. Remarkably, when cells of arterial origin (PAECs) were used to reseed the tissue, only extra-capillary vessels were recellularized. In contrast, areas of lung that originally housed microvascular endothelium remained acellular. The reverse was seen when cells of microvascular origin (MVECs) were used to re-endothelialize the vasculature: cells were observed in microvasculature (capillaries) whereas extra-capillary vessels remained acellular. Interestingly, progenitor enriched endothelial cells (RMEPCs) had tropism for both larger vessel (i.e. extra-capillary) and microvascular (capillary) ECM and showed more matrix coverage when compared to lungs reseeded with PAECs or MVECs alone (Figure 3A). Importantly, RMEPCs displayed attachment within both extra-capillary and capillary vessels when compared to PAECs and MVECs after 8 days of incubation (Figure 3A **)**. RMEPCs displayed widespread expression of VE-Cadherin/CD144–a major protein in establishing membrane permeability (Figure 3B). The detection of VE-Cadherin supports the use of RMEPCs to recreate a functional barrier membrane throughout the decellularized vasculature. Together, the data indicate that endothelial cells derived from the microvascular segments and enriched for progenitors can revascularize all pulmonary segments.

### Revascularized Lung Scaffolds Retain Endothelial Cells that Maintain Functional Attributes

**FIGURE 4:**
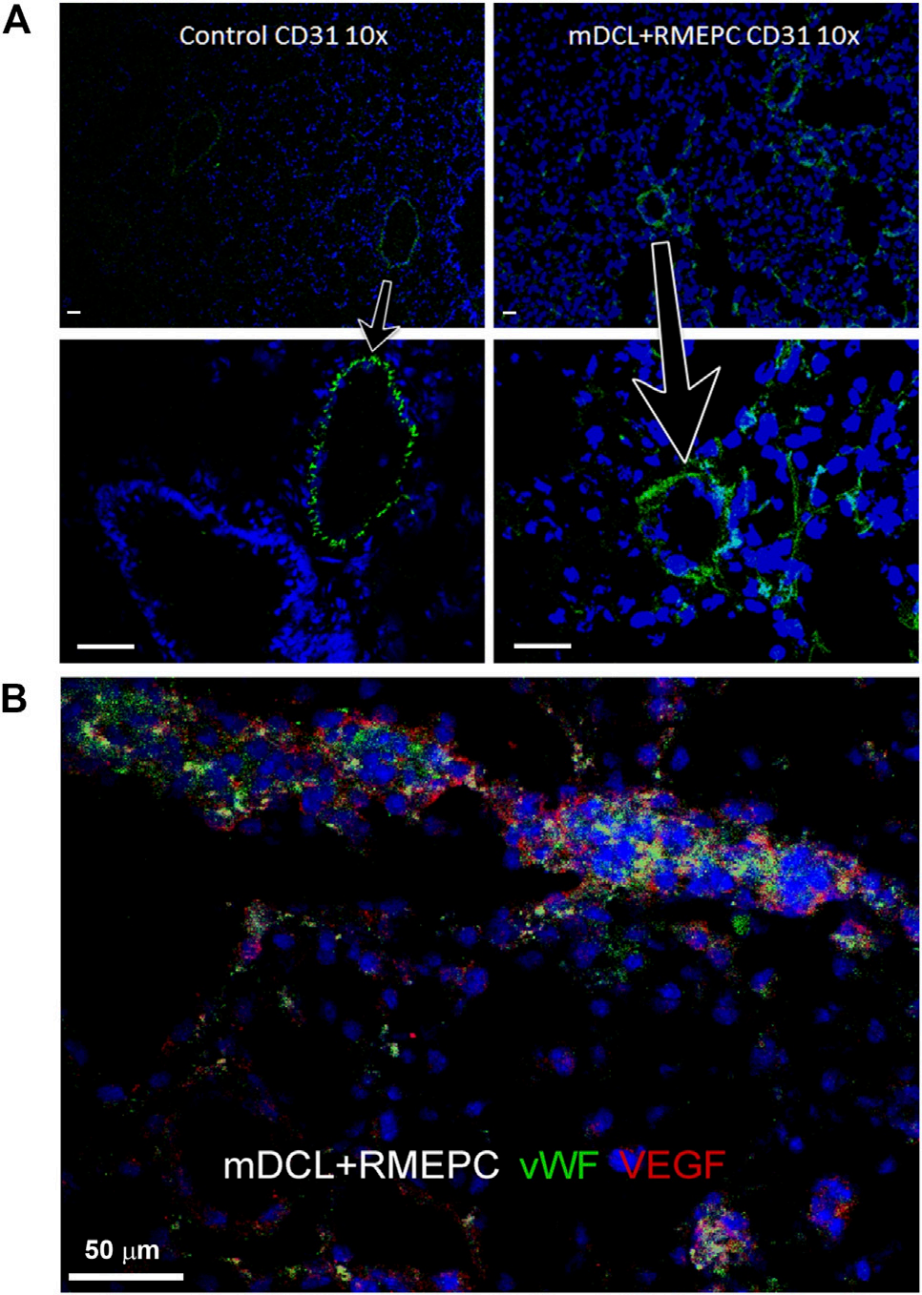
RMEPC express endothelial markers after reseeding on decellularized mouse lungs. **(A)** PECAM-1 (CD31) expression in rat lung control and recellularized lungs. Images taken at ×100 magnification, ×10 objective; DAPI nuclear stain in blue. Arrows point to zoomed areas. **(B)** Immunofluorescence of representative image of RMEPC-recellularized lungs at ×630 magnification (×63 objective) showing expression of von Willebrand factor (vWF) and vascular endothelial growth factor (VEGF). DAPI nuclear stain in blue. Scale bars set to 50 µm in each panel.

In Figure 4A, decellularized mouse lungs reseeded with RMEPCs showed positive staining for PECAM-1 (CD31) and cytosolic vWF and VEGF after 8 days of incubation. This indicates that RMEPC possess the capacity to adopt a mature endothelial phenotype and retaining fundamental molecular cues for their functional behavior. Binding to GSI and HPA lectins could not be used to distinguish endothelial subtypes since these lectins could also bind to decellularized lung ECM (Supplementary Figure S2).

**FIGURE 5:**
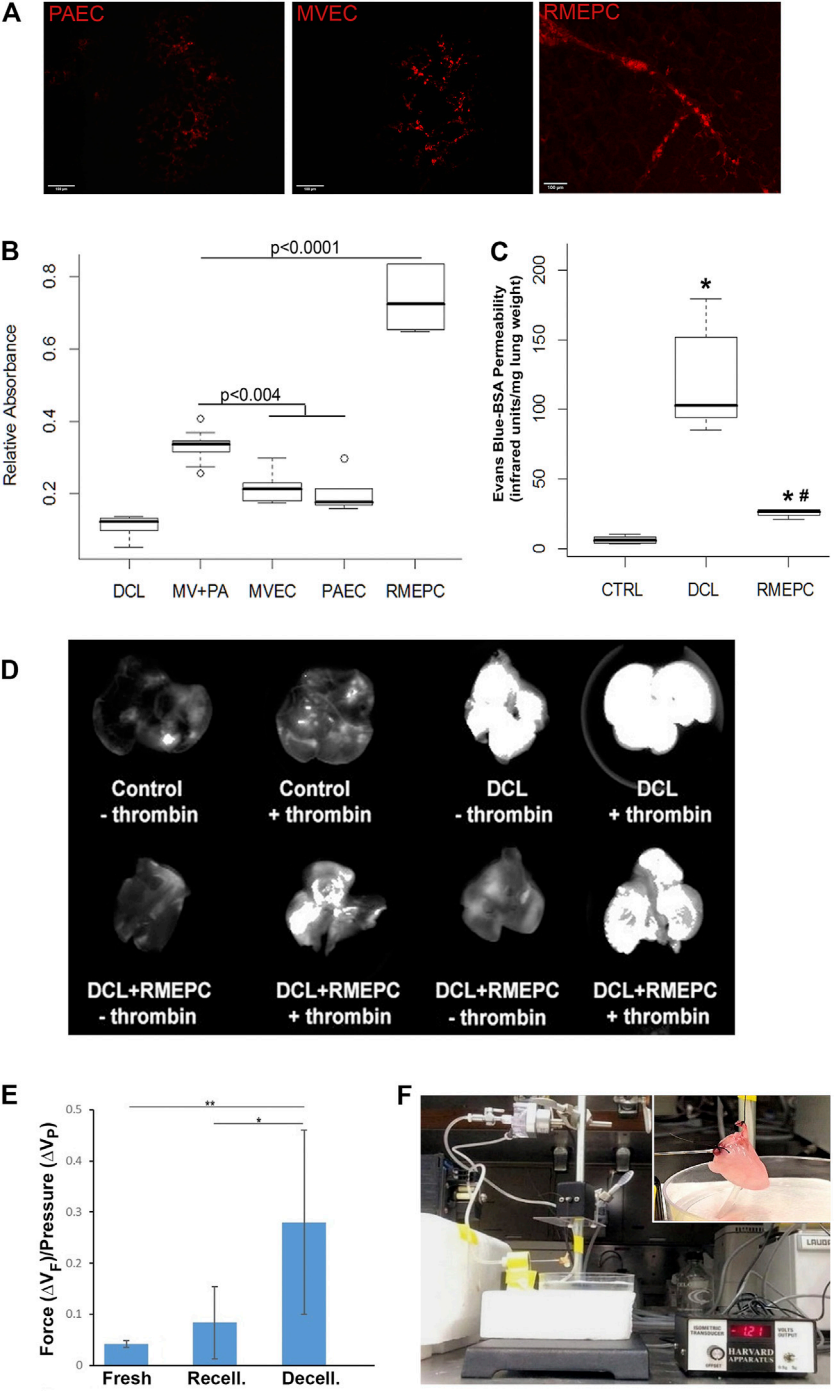
Recellularized lungs are functional and maintain endothelial barrier integrity. **(A)** Uptake of Dil-Ac LDL in revascularized lungs. Cryosections (5 µm) viewed via confocal microscopy (Left–PAEC, Middle–MVEC, Right–RMEPC). **(B)** Thrombomodulin activity assay of recellularized lungs after 45 min perfusion with α-thrombin and protein C colorimetric activity was normalized to whole mouse lung controls (*n* = 6) and compared to decellularized lungs (DCL, *n* = 6), all other groups *n* = 3. **(C)** Vascular permeability of RMEPC-recellularized lungs (*n* = 4) compared to mouse lung controls (CTRL, *n* = 8) and decellularized lungs (DCL, *n* = 8) measured by near-infrared spectrophotometry of perfused Evans-blue-tagged albumin (EB-BSA). *p < 0.0000004 vs. control; *^#^ *p* < 0.0004 vs. decellularized lungs. **(D)** Representative whole lung near-infrared spectrophotometry of EB-BSA showing barrier integrity of RMEPC-recellularized lungs and increased permeability in response to thrombin (+). **(E)** Comparison of the average change in force (∆V_F_) gained per increase in pressure (∆V_P_) for native-fresh (*n* = 4), decellularized (*n* = 7), and RMEPC-recellularized (*n* = 6) mouse lungs (* indicates a *p*-value <0.05; ** indicates a *p*-value < 0.001). **(F)** Setup of isogravimetric experiment and insert of a close-up of heart/lung *en bloc*.

Figure 5A shows that decellularized mouse lungs reseeded and incubated for 7 days with rat PAECs, MVECs, or RMEPCs could take up Acetylated LDL. Thrombomodulin activity, relative to non-decellularized mouse lung controls (Figure 5B), was significantly increased in decellularized mouse lungs reseeded with RMEPCs compared with lungs reseeded with MVECs or PAECs or a combination of MVEC and PAECs (MV + PA). Collectively, these data demonstrate the extraordinary capacity of RMEPCs to generate endothelium barriers with functional behaviors.

### RMEPCs-reseeded decellularized lung scaffolds display enhanced repopulation and functional barrier properties

To demonstrate that decellularized lungs reseeded with rat RMEPCs could maintain a functional barrier, lungs (after 8 days incubation) were perfused with EB-tagged BSA for 5 min. After clearing the vasculature with PBS, lungs were imaged for near-infrared fluorescence that can detect any albumin-conjugated-Evans Blue dye leaking into the pulmonary interstitium and airway space. As shown in Figure 5C, intact control lungs displayed marginal vascular leak as evidenced by the low detection of extra-vascular albumin. In contrast, decellularized lungs exhibited substantial vascular leak as evidenced by the high levels of extra-vascular albumin detected. Decellularized lungs reseeded with RMEPCs demonstrated low vascular leak approximating that exhibited by intact control lungs, indicating significant re-endothelialization of the lung scaffold. Importantly, Figure 5D shows that lungs reseeded with RMEPCs respond to α-thrombin with an increase in vascular permeability, albeit higher than the response of intact normal lungs. Therefore, lungs re-endothelialized with RMEPCs not only generated barriers that restrict the movement of fluid and solutes from the vascular to the interstitial space, but formed a barrier that responds to a circulating agonist known to increase permeability.

**FIGURE 6:**
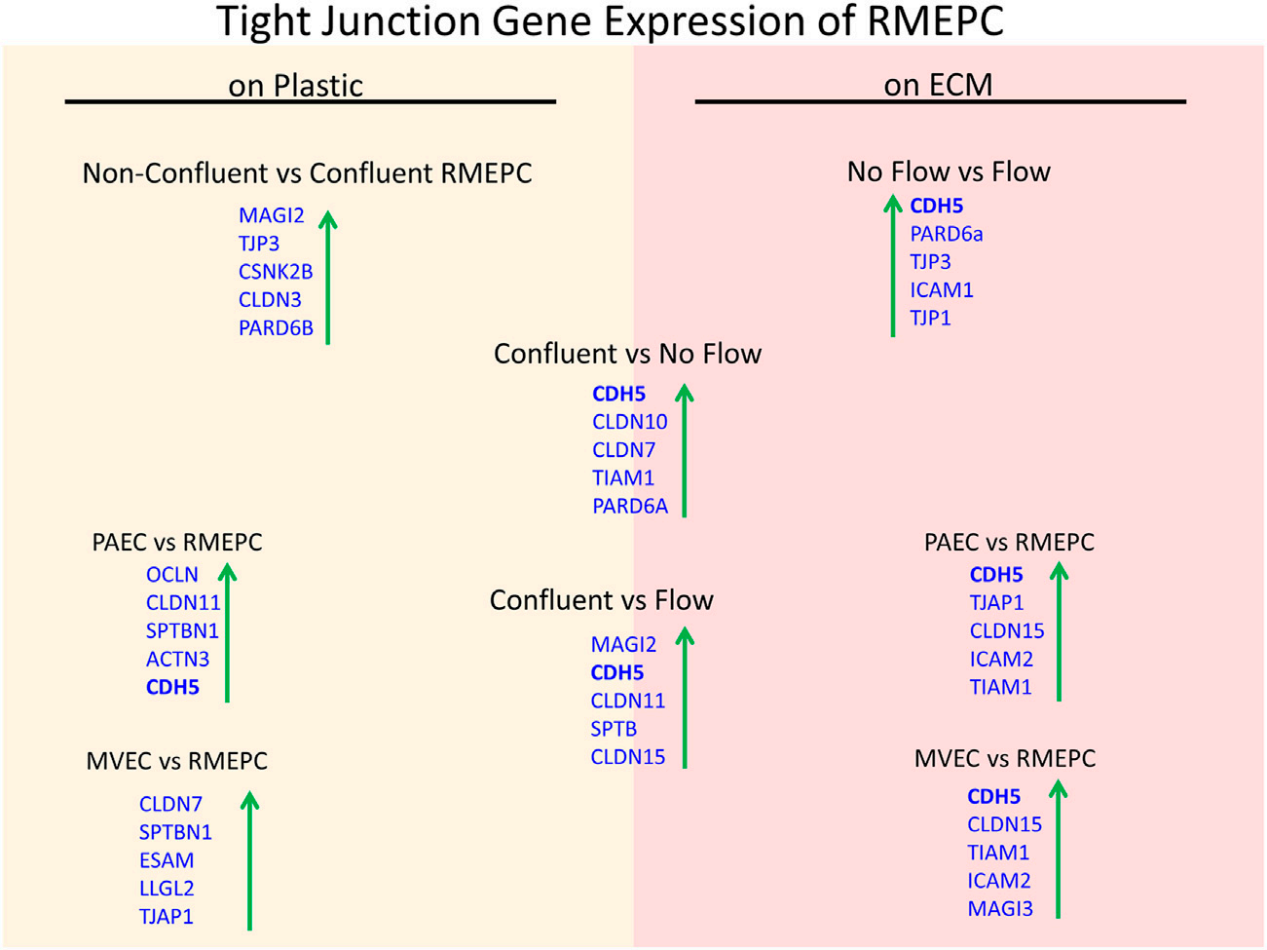
Confluency, extracellular matrix and no-flow conditions promote the highest expression of tight junction genes by RMEPCs. Top left shows the top upregulated tight junction genes in response to confluency on RMEPCs cultured on plastic. Bottom left shows the increase in genes in confluent RMEPCs compared to confluent PAECs and MVECs cultured on plastic. The right-hand side (red) shows day 7 gene expression when seeded by perfusion on decellularized mouse lung ECM. The bottom right shows the top tight junction genes upregulated in RMEPCs vs. PAECs and MVECs on ECM (with flow) after 7 days. The middle portion shows the increases in tight junction genes in RMEPC in conditions vascular flow and no-flow for 7 days compared to confluency on plastic. The far top right compares no-flow vs. flow of RMEPCs on ECM.

**FIGURE 7:**
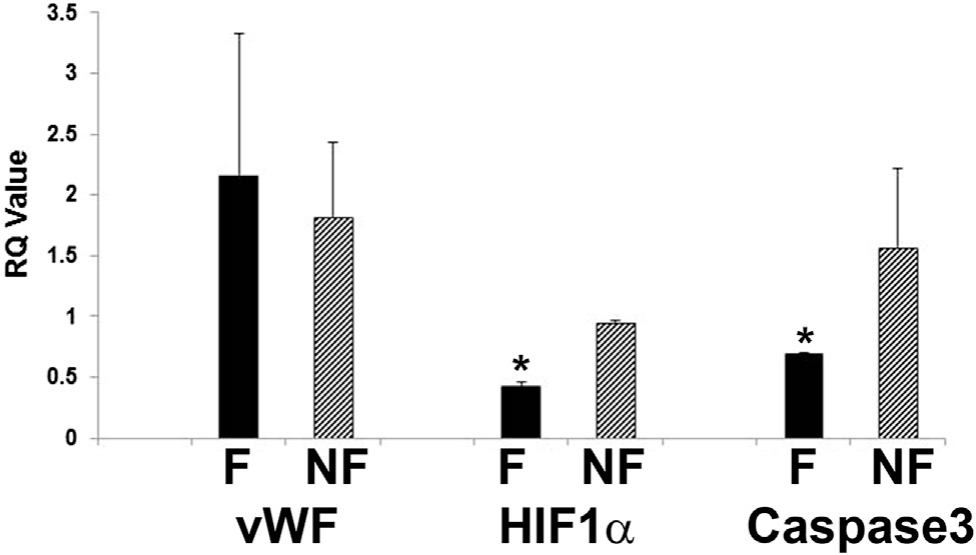
Decreased apoptosis and hypoxic signal in RMEPC on ECM after 1 week in flow conditions. qRT-PCR for vWF, HIF-1a and caspase-3 are shown. F = flow, NF = no flow. *p=<0.05 for F vs. NF (*n* = 3/group).

Further evidence of tight barrier formation was obtained by measuring expression of tight junction and cell adhesion genes by qRT-PCR. Comparisons were made for RMEPCs grown to confluence vs. non-confluence in 2D tissue culture, those grown on decellularized lung ECM scaffolds versus 2D tissue culture, and versus rat PAEC and MVEC cells in 2D and on decellularized lung ECM in pulsatile flow conditions. Figure 6 and Supplementary Table S1 show the top upregulated mRNAs in RMEPCs grown to confluence on plastic, with the highest being MAGI2, a gene that encodes for membrane-associated guanylate kinase inverted two protein. MAGI2 was further upregulated when RMEPC were grown on decellularized lungs under flow conditions. Growth on lung ECM also caused upregulation of several claudins as well as cadherin 5 (Supplementary Tables S2, S3). Since endothelial cells also respond to fluid flow and shear stress, mRNA expression of RMEPCs reseeded in decellularized lungs were compared in conditions of flow vs. no-flow (Supplementary Table S4). In flow conditions, expression of many tight junction genes was downregulated compared to no-flow conditions (i.e. they were higher in no-flow), a finding that is consistent with the formation of mature junctions *in vivo*. Figure 7 shows that in conditions of flow, expression of HIF-1a and caspase-3 mRNAs were decreased compared to no-flow conditions when RMEPCs were seeded on decellularized ECM, indicating that flow decreases apoptosis and hypoxia in the endothelial cells as expected.

**FIGURE 8:**
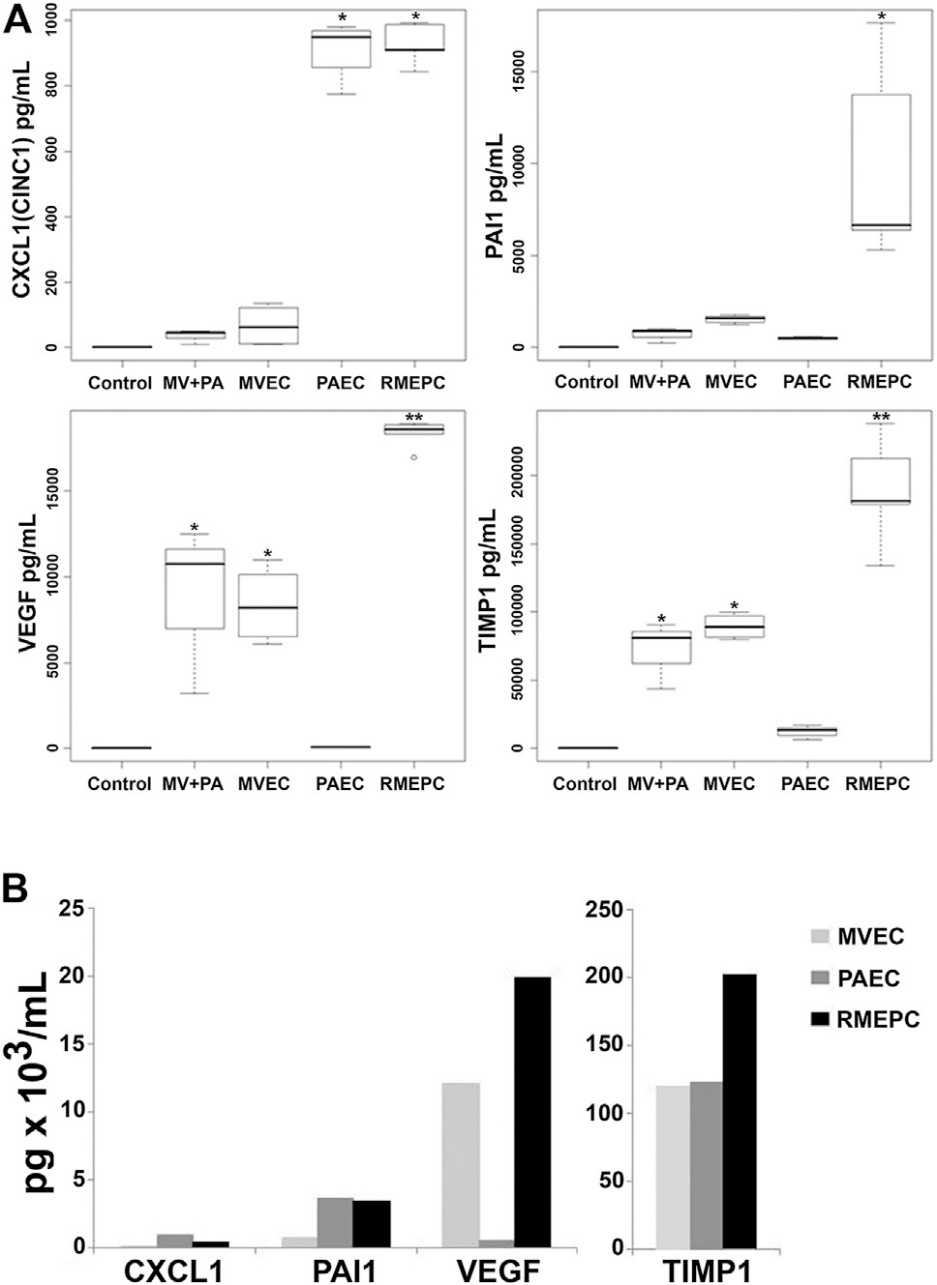
**(A)** Secretion of endothelial cell mediators from perfusate of decellularized lungs reseeded with RMEPC, MVEC, PAEC, or both MVEC and PAEC (“MV + PA”). “Control” indicates complete culture medium alone. Perfusates were sampled from reseeded constructs at 48 h post-seeding. * Single asterisk indicates *p* < 0.05 versus non asterisk, and double asterisk (**) groups. For all samples, *n* = 8, except for RMEPC where *n* = 10. **(B)** *In vitro* production of endothelial cell mediators after 48 h of 2D tissue culture in flasks. These single samples were run in duplicate with CV < 15%.

To further evaluate the function of reseeded cells, effluents from PAEC-, MVEC- or RMEPC-infused matrices were examined for secreted vascular factors. Figure 8A shows that RMEPC-seeded matrices secreted higher levels of PAI-1, TIMP-1 and VEGF-A compared to those seeded with MVECs, PAECs, or MVECs + PAECs. RMEPC-seeded lung scaffolds secreted levels of CXCL1 similar to PAEC-seeded scaffolds and were higher than those seeded with MVECs or MVECs + PAECs. Analysis of cell culture supernatants of these cells grown in tissue culture flasks (Figure 8B) showed that, within 2D culture, RMEPCs had the highest production of VEGF-A and TIMP-1, while levels of PAI-1 and CXCL1 were similar between RMEPCS and PAECs (and higher than MVECs). The discrepancies seen between cells grown in culture flasks versus those in reseeded lung scaffolds highlight the effect of ECM on signaling cytokine production in the three endothelial cell populations.

## Discussion

We have described a pulmonary endothelial cell population generated from microvasculature that can functionally recellularize heterogeneous vascular segments of decellularized lung scaffolds. Perfusion of the vasculature with a pulsatile flow of endothelial populations enriched with progenitor cells assembles functional pulmonary vasculatures using a xenogeneic reseeding approach. Moreover, infused mature pulmonary microvascular endothelial cells consistently display selective tropism for their vascular segment of origin, whereas PAECs prefer to repopulate larger vessel conduits. Along with work conducted by other groups (Marcu et al., 2018; Scarritt et al., 2018), this observation indicates that engraftment into specific vascular segments is dependent on the endothelial cell phenotype when using mature endothelial cells. Importantly, infused endothelial progenitor cells were stable for up to 10 days’ post initial infusion and, under the flow conditions utilized, they retained functional endothelial attributes and developed functional restrictive barriers. Our findings provide the basis necessary for developing functional, autologous, transplantable lungs capable of gas exchange, proper hemodynamics, and incorporation into the host’s circulatory system without thrombogenicity. In addition, the phenotypic characterization of cells capable of such re-endothelialization could be useful for identifying endothelial progenitors suitable for efficient vascular organ and tissue engineering, regeneration and repair.

Endothelial cells lining the pulmonary arteries, veins, and alveolar capillaries exhibit remarkable heterogeneity both in structure and in function. Ultrastructural analysis indicate that endothelial cells derived from the pulmonary artery (PAECs) reside in thicker basement membranes, display a bulging-like phenotype and possess cell to cell borders that are clearly distinguishable (King et al., 2004; Aird, 2012; Przysinda et al., 2020). In contrast, pulmonary microvascular endothelial cells (MVECs) reside in thinner basement membranes, display a flatter phenotype, and overlap at the border with nearby cells forming what appear to be stronger junctional complexes when compared to their conduit vessel-residing relatives (King et al., 2004; Aird, 2012). The structural differences are accompanied by significant differences in stress responses most of which have been studied in the context of inflammation and barrier restrictive properties. The clearly identifiable heterogeneity existing within the pulmonary circulation has been attributed to both environmental factors and epigenetic modifications. Exposure to high PCO_2_, low PO_2_ and relatively higher vascular pressure (when compared to other pulmonary vascular segments) demand unique adaptive processes in the endothelium lining pulmonary arteries while reciprocal exposures are seen in microvascular regions where gas exchange occurs (Aird, 2007). In addition, several epigenetic imprints have been described, in particular those which explain, at least in part, the enhanced replication competence of the microvascular endothelial cells when compared to their conduit-derived pulmonary artery counterparts (Alvarez et al., 2008; Clark et al., 2008).

Populations of resident endothelial cells enriched with progenitor cells have been previously described in the pulmonary circulation (RMVECs) (Alvarez et al., 2008). Compared to PAECs and MVECs, RMEPCs have longer telomeres, and a higher proliferative capacity without evidence of transformation transelectrical membrane resistance. Furthermore, they exhibited the highest transepithelial membrane resistance (Alvarez et al., 2008). Our results demonstrated that those progenitor cells do not exhibit selective tropic abilities as shown by PAECs or MVECs but display a capacity to seed in any vascular segment. Considering our use of similar perfusion conditions, the data indicates that unique physical properties of the cells, specific expression of integrins, or other matrix-recognizing molecules are determinants for recognition of distinctive scaffold components that lure cells towards specific segments of the circulation. Similar work conducted with PAECs and MVECs, as well with HUVECs and pulmonary venous ECs, has shown similar limitations in vascular coverage and the need for combination seeding in order to achieve maximal coverage, including distal areas (Ren et al., 2015; Gilpin et al., 2016; Scarritt et al., 2018). Importantly, RMEPCs displayed an enhanced replicative competence (Alvarez et al., 2008) and/or enhanced adhesive ability which explains their remarkable vascular repopulation. Consequently, infusion of RMEPCs under the flow conditions described was able to generate a restrictive barrier with limited permeability for large solutes and detectable VE-cadherin, a major endothelial adhesion molecule, without the need for combination seeding.

Flow-seeding technology is necessary to perfuse the complete vasculature of decellularized whole organs (Walluscheck et al., 1996). Flow also induces physiological shear stress responses, which induces endothelium to shift from cobblestone morphology to align with the direction of flow, stabilizing endothelial-endothelial cell communications and promoting remodeling of focal adhesion sites (Davies et al., 1994; Malek and Izumo, 1996; Chiu and Chien, 2011; Adamson et al., 2013). While this technology is necessary for restoration of functional vasculature, there are numerous obstacles that are associated with seeding cells within acellular tissue and should be consider by any research group studying tissue decellularization or recellularization. Harsh decellularization as well as improper storage (i.e. freezing, thawing) of acellular scaffolds can lead to disruption of components and architecture (Calle et al., 2016; Feng et al., 2020). During cell seeding of pulmonary vasculature, positioning of the scaffold (supine vs. prone vs. upright) and the physiological distribution of blood flow within the pulmonary vascular tree greatly affect distribution of cells within lobes (Stabler et al., 2016). In order to prevent clots from disrupting complete organ decellularization and subsequent vascular recellularization, anticoagulants and thrombolytics are commonly employed to prevent and dissolve clots, respectively (Price et al., 2015; Doi et al., 2017). The method of seeding is another crucial factor in achieving high vascular coverage within acellular scaffolds. Recent reviews have stated the importance of using low cellular concentrations within large volumes, choosing gravity-dependent infusion over pump-dependent infusion, as well as seeding cells with both antegrade and retrograde perfusion to attain greater coverage throughout the entire vasculature of the acellular scaffold (Ohata and Ott, 2000; Robertson et al., 2014; Leiby et al., 2020). While we used pump-driven seeding in only the antegrade direction, we did not experience the seeding issues described above. This might be explained by our inclusion of priming the scaffolds with 24 h each of saline and media alone, and filtering of cell suspensions prior to reseeding to prevent clumping.

Our data show that, under continuous pulsatile flow, a significant proportion of RMEPCs was able to form a stable barrier for up to 8 days post initial infusion. Another benefit in the use of flow–perfusing conditions is the removal of any potentially thrombogenic gas nuclei within the lung tissue, as hydrostatic pressure has been shown to reduce the thrombogenicity of polytetrafluoroethylene vascular grafts (Ritter et al., 1989). Our data suggest that using native lung scaffolds as a substrate for cell attachment provides sufficient binding to withstand pulsatile flow through the vasculature. We recognize that further hemodynamic studies are necessary for determining the optimal infusion conditions (rate, time, pressures) to obtain a pulmonary barrier similar to that observed *in vivo*.

Our results demonstrate that endothelial heterogeneity is required for bioengineering functional vasculature, consistent with *in vivo* findings (Belloni and Tressler, 1990; Sieminski et al., 2005; Verhamme and Hoylaerts, 2006; Stevens, 2011; Scarritt et al., 2018). All endothelial cells used for this study were able to attach and function on decellularized lung matrices, although RMEPC showed significantly greater attachment, maintenance of functional endothelial attributes (e.g. thrombomodulin activity, VEGF production), barrier restrictive properties, and a reduction of apoptotic signals in the presence of flow. Interestingly, RMEPC showed less CD31 expression than PAEC or MVEC *in vitro*. This may be explained, in part, by the heterogeneous nature of this particular primary endothelial lineage, which is enriched with progenitor cells not yet expressing migration adhesion molecules. In fact, among the tight junction and adhesion molecules we looked into (see Supplementary Table S3), there were only a few that had a relatively higher gene expression within our RMEPCs. RMEPC have enhanced adherence to and maturation within multiple regions compared to PAEC and MVEC, apparently without much need for CD31 as seen in Figure 2. These data collectively lend evidence toward utilizing a single source-derived endothelial population that, after enrichment with progenitor cells, favors revascularization of each segment of the pulmonary circulation.

The qPCR results indicate that there are differences in the expression of genes for LDL-receptor and selected scavenger receptors (other proteins that play a role in the uptake of LDL) amongst the 3 cell types we studied. Based on information from public databases created from compiled murine endothelial cell transcriptomes (Goveia et al., 2020; Kalucka et al., 2020), it was expected that similar expressions of genes for LDL-receptor, Scarf1, and Scarf2 would be found since LDL is an important nutrient for endothelial cells, and the class F scavenger receptors are classified as endothelial-specific (Patten, 2018). While public databases show a mixed expression of genes for αv, β1, β2, and β5 among all endothelial cell types, we found that the PAECs had relatively higher expression of all but β2, whereas our RMEPCs had the highest expression of β2, an integrin that forms adhesion proteins by binding to CD11 a-d and primarily seen amongst leukocytes (Fagerholm et al., 2019). While outside the scope of this study, more work is needed in both understanding the expression of these important proteins as they will further our understanding of supplying cells with proper nutrients during recellularization and insight into what sites they will be able to adhere to during the seeding step of regenerating tissue.

While differential tropism might indicate a difference in expression of integrins responsible for attachment to the ECM, we do not yet understand the molecular determinants that resulted in the endothelial cell recognition of specific vascular segments within the pulmonary circulation. Canonical homo- and hetero-topic interactions are ascribed to the engagement of integrins and the contribution of the endothelial cell glycocalyx. Based on our flow cytometry data of our markers of interest, there is little differential expression of cell surface adhesion molecules, though we were able to detect P-selectin, which is important for ECs and platelets during an inflammatory response. While GAGs play a role in cell adhesion to implantable grafts (Ibrahim and Ramamurthi, 2008), they may also allow for anatomically relevant attachment to decellularized native ECM, by providing native geospatial cues. This could be facilitated by glycosphingolipids, glycoproteins, or proteoglycans, which have all been documented as modulators of cell localization. If so, these terminal sugar residues would shift cell-matrix binding toward common glycosylation motifs, instead of canonical integrin/collagen or laminin binding protein (LBP) within focal adhesions (that GAGs also modulate by conferring steric hindrance). Differential expression of saccharide-binding receptors, such as P-selectin, may explain the increased binding of the RMEPC. Alternatively, binding domains within ECM proteins favor a particular endothelial glycocalyx. Growth factors, cytokines, and chemokines trapped within native matrix may impact origin-specific endothelial cell-matrix binding. As with most physiological systems, there is likely a dynamic balance between these influences on cell adhesion and subsequent intracellular signal transduction through anchor proteins (Burgess et al., 2006; Schultz and Wysocki, 2009; Ishihara et al., 2018; Mammoto and Mammoto, 2019).

In summary, we have identified a single cell source that is capable of re-endothelializing the lung in a comprehensive manner providing a functional barrier. Furthermore, we have determined that endothelial cells that exhibit selective tropism are suboptimal for functional re-endothelialization of acellular lungs. Along with recent studies published by others we conclude that it is vital to include endothelial progenitors, such as RMEPCs, in the seeding process of decellularized lung scaffolds in order to improve endothelial function, barrier permeability, and coverage throughout the entire pulmonary vasculature (Ren et al., 2015; Nagao et al., 2016; Scarritt et al., 2018). Although future studies will help us understand the mechanism of the stochastic nature of lung microvascular EPCs in recellularizing the lung vascular extracellular matrix, it is apparent that our findings warrant moving ahead on determining their translational capacity for bioengineering, therapeutic applications, and understanding regeneration and repair for lungs and other vascularized organs.

## Data Availability

The original contributions presented in the study are included in the article/Supplementary Materials, further inquiries can be directed to the corresponding author.
