# Supplementary material for: Engineering Functional Vasculature in Decellularized Lungs Depends on Comprehensive Endothelial Cell Tropism
Source: Front Bioeng Biotechnol. 2021 Aug 16;9:727869. doi: 10.3389/fbioe.2021.727869 (PMC8415401; doi:10.3389/fbioe.2021.727869)
Supplement: Supplementary file 1 [file DataSheet1.PDF]

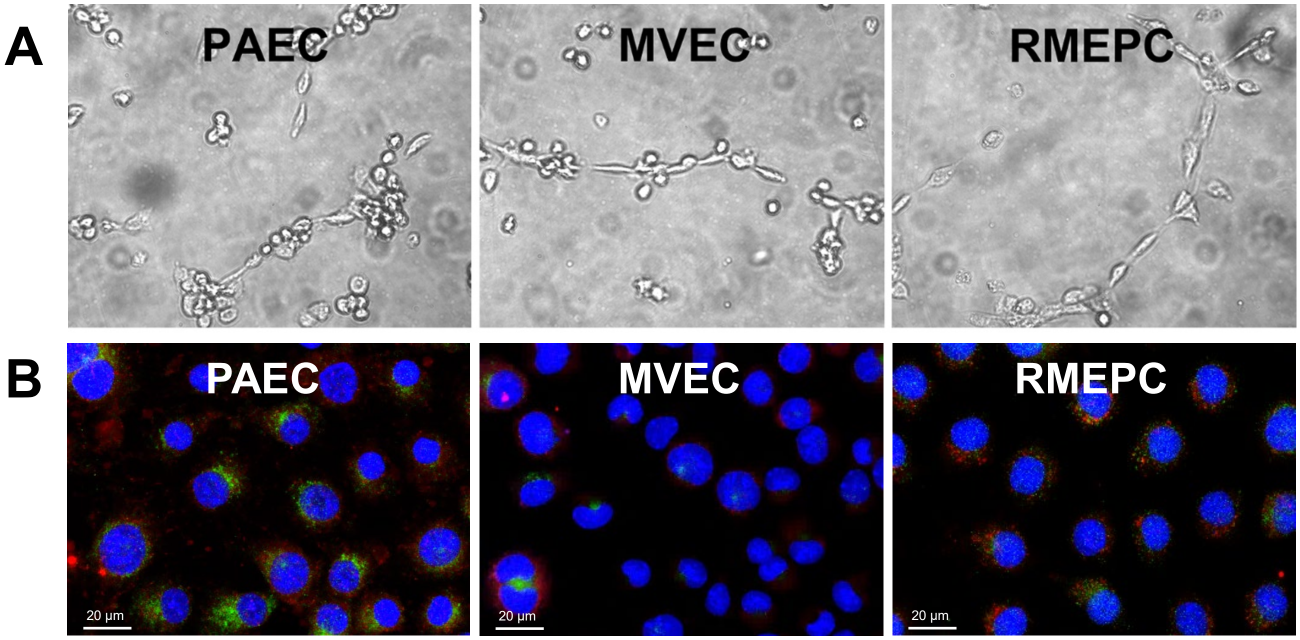

Supplemental Figure 1. A: *Tube formation*. Cultured rat endothelial cells seeded onto tissue culture plates coated with 100µl/cm^2 Matrigel showed capillary network-forming potential as early as four hours post-seeding. Photographs pictured above were taken at 8 hours post-seeding at 100X magnification (10x objective lens). **B**: *LDL-Uptake*. Ac-LDL uptake in vitro. Images were taken after overnight starvation and 4 hours of incubation with modified LDL at 400X magnification (40x objective lens). Blue: Nuclei; Green: VE-Cadherin; Red: Dil-acetylated-LDL. Scale bar = 20µm.

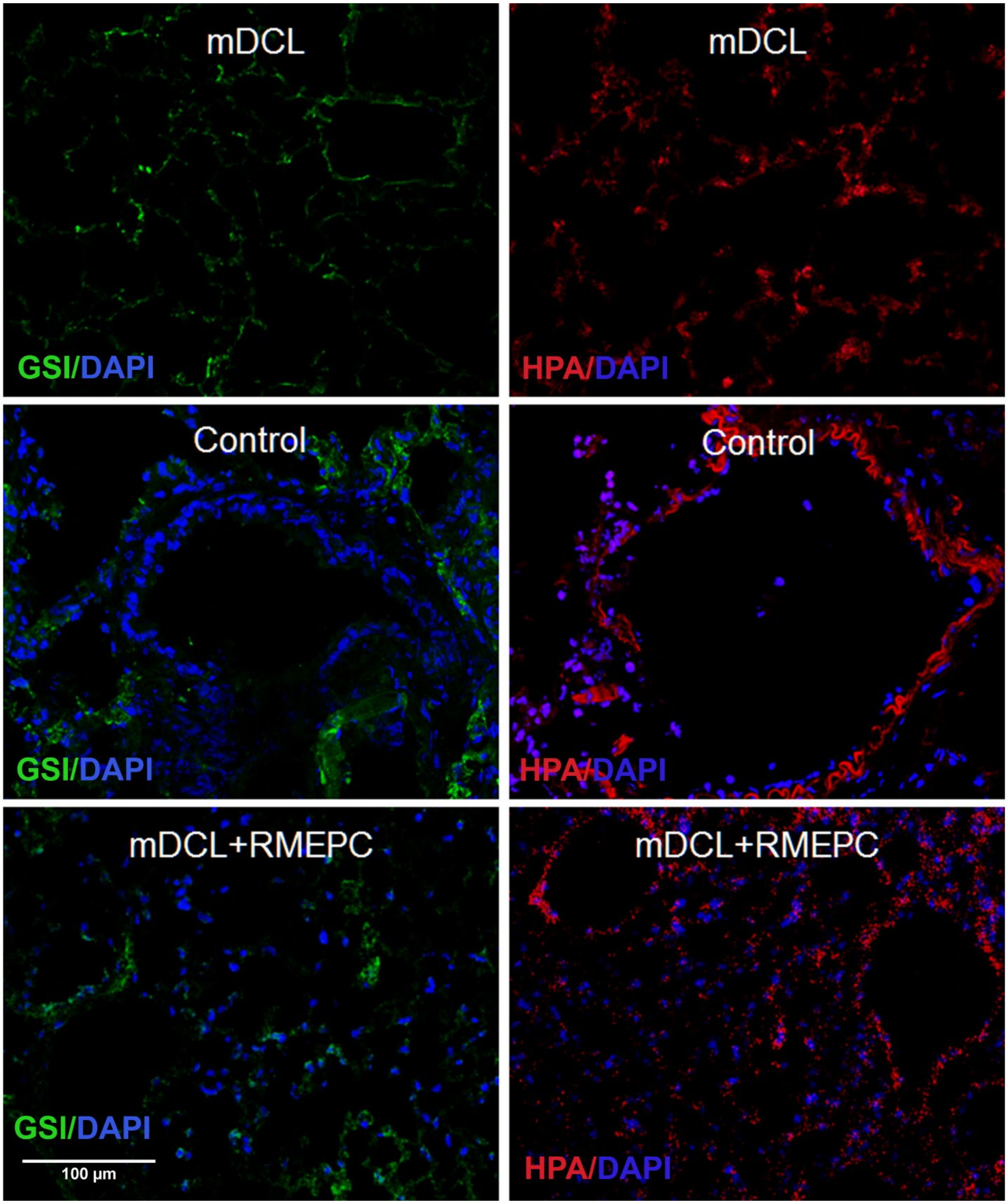

Supplemental Figure 2. Confocal microscopy of typical Griffonia simplicifolia (GSI, green) and Helix pomatia (HPA, red) lectin binding patterns seen in control rat lungs compared with decellularized and RMEPC-recellularized mouse lungs at 200x magnification (20X objective lens). DAPI fluorescence was used in all images to visualize cell nuclei. Scale bar = 100 µm.

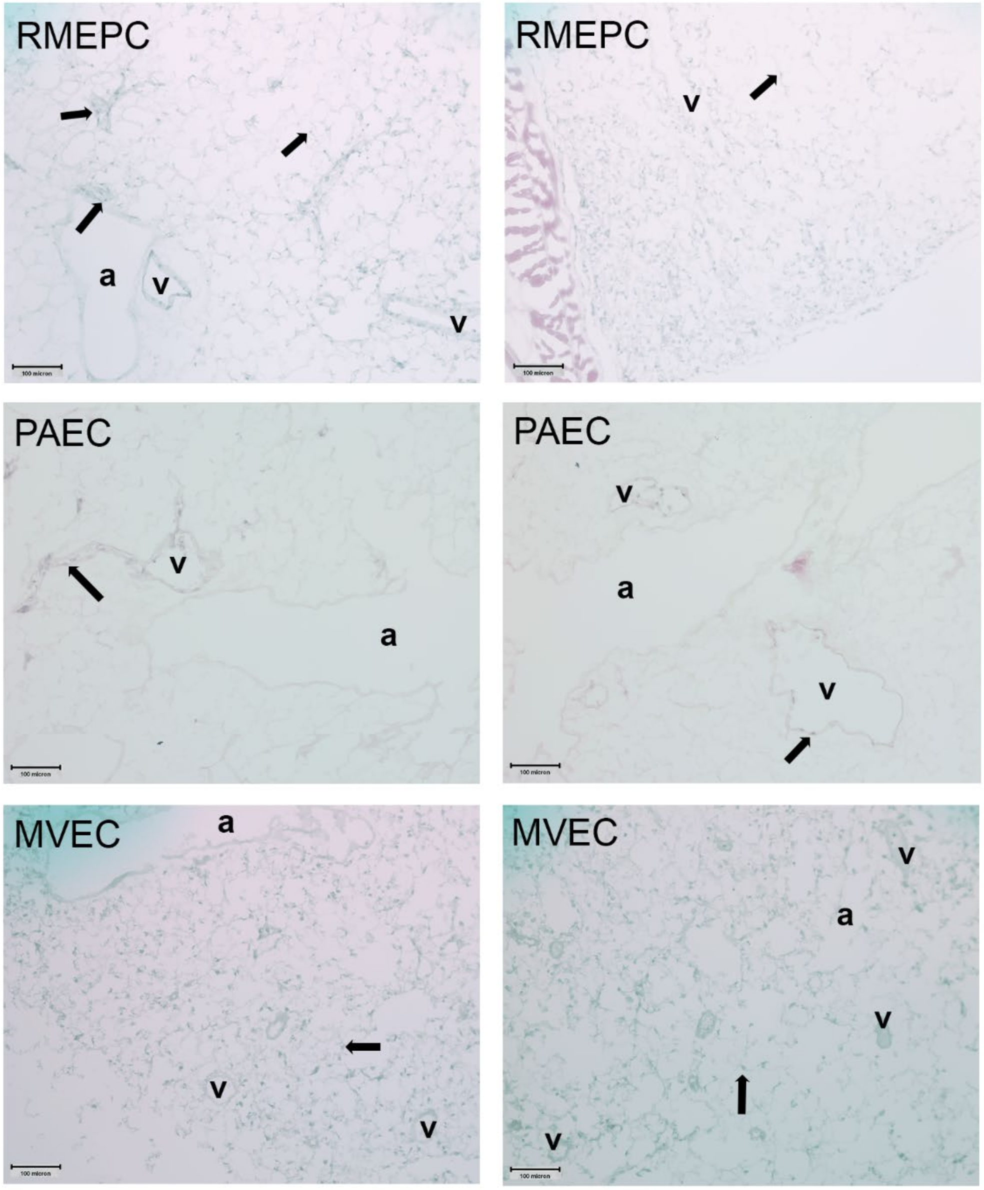

Supplemental Figure 3. Additional H&E-stained cryosections of decellularized whole lungs re-seeded with either RMEPCs (top row), PAECs (middle row), or MVECs (bottom row). Images taken at 100X magnification (10X objective lens), with airways (a) and vessels (v) marked. Some examples of cells are indicated by arrows. Scale bar = 100µm.

Supplemental Table 1: Tight junction gene expression of RMEPC confluence on plastic vs non-confluence on plastic. Green=upregulation with confluence, red=downregulation with confluence

Symbol	Entrez Gene Name	Location	Plastic (confluent) vs Plastic (not confluent)
MAGI2	membrane associated guanylate kinase, WW and PDZ domain containing 2	Plasma Membrane	3.490
Tjp3	tight junction protein 3	Plasma Membrane	2.820
CSNK2B	casein kinase 2, beta polypeptide	Cytoplasm	2.440
CLDN3	claudin 3	Plasma Membrane	2.190
PARD6B	par-6 family cell polarity regulator beta	Plasma Membrane	2.130
ACTN1	actinin, alpha 1	Cytoplasm	1.940
LLGL1	lethal giant larvae homolog 1 (Drosophila)	Cytoplasm	1.840
CLDN11	claudin 11	Plasma Membrane	1.800
RHOA	ras homolog family member A	Cytoplasm	1.630
SMURF1	SMAD specific E3 ubiquitin protein ligase 1	Cytoplasm	1.620
LLGL2	lethal giant larvae homolog 2 (Drosophila)	Cytoplasm	1.600
TJAP1	tight junction associated protein 1 (peripheral)	Plasma Membrane	1.520
GSK3A	glycogen synthase kinase 3 alpha	Nucleus	1.480
F11R	F11 receptor	Plasma Membrane	1.400
CSNK2A2	casein kinase 2, alpha prime polypeptide	Cytoplasm	1.390
CDH5	cadherin 5, type 2 (vascular endothelium)	Plasma Membrane	1.370
SPTB	spectrin, beta, erythrocytic	Plasma Membrane	1.260
SYMPK	symplekin	Cytoplasm	1.240
CLDN18	claudin 18	Plasma Membrane	1.180
CTTN	cortactin	Plasma Membrane	1.160
YBX3	Y box binding protein 3	Nucleus	1.150
CLDN12	claudin 12	Plasma Membrane	1.140
ICAM1	intercellular adhesion molecule 1	Plasma Membrane	1.140
Cdk4	cyclin-dependent kinase 4	Nucleus	1.110
SPTBN2	spectrin, beta, non-erythrocytic 2	Cytoplasm	1.080
ILK	integrin-linked kinase	Plasma Membrane	1.050
ACTN4	actinin, alpha 4	Cytoplasm	1.030
TJP2	tight junction protein 2	Plasma Membrane	1.020
RAC1	ras-related C3 botulinum toxin substrate 1 (rho family, small GTP binding protein Rac1)	Plasma Membrane	-1.040
PARD6A	par-6 family cell polarity regulator alpha	Plasma Membrane	-1.100
GNAI2	guanine nucleotide binding protein (G protein), alpha inhibiting activity polypeptide 2	Plasma Membrane	-1.130
ICAM2	intercellular adhesion molecule 2	Plasma Membrane	-1.130

AMOTL1	angiomotin like 1	Plasma Membrane	-1.150
CDC42	cell division cycle 42	Cytoplasm	-1.250
CTNNB1	catenin (cadherin-associated protein), beta 1, 88kDa	Nucleus	-1.280
CSNK2A1	casein kinase 2, alpha 1 polypeptide	Cytoplasm	-1.470
ARHGEF2	Rho/Rac guanine nucleotide exchange factor (GEF) 2	Cytoplasm	-1.490
CLDN15	claudin 15	Plasma Membrane	-1.510
MARK2	MAP/microtubule affinity-regulating kinase 2	Cytoplasm	-1.590
CLDN19	claudin 19	Plasma Membrane	-1.620
VAPA	VAMP (vesicle-associated membrane protein)-associated protein A, 33kDa	Plasma Membrane	-1.630
TIAM1	T-cell lymphoma invasion and metastasis 1	Cytoplasm	-1.660
JAM3	junctional adhesion molecule 3	Plasma Membrane	-1.670
GSK3B	glycogen synthase kinase 3 beta	Nucleus	-1.730
PARD3	par-3 family cell polarity regulator	Plasma Membrane	-1.880
PRKCI	protein kinase C, iota	Cytoplasm	-2.170
EPB41L1	erythrocyte membrane protein band 4.1-like 1	Plasma Membrane	-2.810
MLLT4	myeloid/lymphoid or mixed-lineage leukemia (trithorax homolog, Drosophila); translocated to, 4	Nucleus	-2.890
MAGI3	membrane associated guanylate kinase, WW and PDZ domain containing 3	Cytoplasm	-2.930
SPTAN1	spectrin, alpha, non-erythrocytic 1	Plasma Membrane	-3.120
MPP5	membrane protein, palmitoylated 5 (MAGUK p55 subfamily member 5)	Plasma Membrane	-3.400
CASK	calcium/calmodulin-dependent serine protein kinase (MAGUK family)	Plasma Membrane	-3.750
ASH1L	ash1 (absent, small, or homeotic)-like (Drosophila)	Nucleus	-4.030
PTEN	phosphatase and tensin homolog	Cytoplasm	-4.080
SPTBN1	spectrin, beta, non-erythrocytic 1	Plasma Membrane	-4.120
TJP1	tight junction protein 1	Plasma Membrane	-4.560
MPDZ	multiple PDZ domain protein	Plasma Membrane	-5.950

Supplemental Table 2: Tight junction gene expression of RMEPC on lung ECM with no-flow vs confluence on plastic. Green=upregulation on ECM, red=downregulation on ECM

Symbol	Description	Fold Regulation
Cdh5	Cadherin 5	7695.13
Cldn10	Claudin 10	130.53
Cldn7	Claudin 7	104.90
Tiam1	T-cell lymphoma invasion and metastasis 1	102.48
Pard6a	Par-6 (partitioning defective 6,) homolog alpha (C. elegans)	48.12
Tjap1	Tight junction associated protein 1	40.09
Sptb	Spectrin, beta, erythrocytic	36.50
Cldn19	Claudin 19	36.23
Ocln	Occludin	33.43
Cldn18	Claudin 18	27.84
Cldn15	Claudin 15	20.36
Csnk2a1	Casein kinase 2, alpha 1 polypeptide	19.26
Icam1	Intercellular adhesion molecule 1	16.01
Actn4	Actinin alpha 4	11.97
Prkci	Protein kinase C, iota	11.94
Cdk4	Cyclin-dependent kinase 4	11.26
Cldn3	Claudin 3	9.74
F11r	F11 receptor	9.04
Amotl1	Angiomotin-like 1	8.86
Ctnnb1	Catenin (cadherin associated protein), beta 1	6.90
Sympk	Symplekin	5.83
MLlt4	Myeloid/lymphoid or mixed-lineage leukemia (trithorax homolog, Drosophila); translocated to, 4	5.04
Tjp1	Tight junction protein 1	4.43
Cttn	Cortactin	3.85
Pard6b	Par-6 (partitioning defective 6) homolog beta (C. elegans)	3.83
Magi3	Membrane associated guanylate kinase, WW and PDZ domain containing 3	3.70
Gsk3a	Glycogen synthase kinase 3 alpha	3.63
Jam3	Junctional adhesion molecule 3	3.56
Pard3	Par-3 (partitioning defective 3) homolog (C. elegans)	3.39
Ash1l	Ash1 (absent, small, or homeotic)-like (Drosophila)	3.14
Sptan1	Spectrin, alpha, non-erythrocytic 1	3.10
Gnai2	Guanine nucleotide binding protein (G protein), alpha inhibiting 2	3.09
Mark2	MAP/microtubule affinity-regulating kinase 2	2.83
Sptbn1	Spectrin, beta, non-erythrocytic 1	2.69

Ybx3	Cold shock domain protein A	2.10
Epb41l1	Erythrocyte membrane protein band 4.1-like 1	2.07
Cask	Calcium/calmodulin-dependent serine protein kinase (MAGUK family)	2.01
Smurf1	Similar to RIKEN cDNA 4930431E10	1.90
Cldn12	Claudin 12	1.78
Mpp5	Membrane protein, palmitoylated 5 (MAGUK p55 subfamily member 5)	1.75
Csnk2a2	Casein kinase 2, alpha prime polypeptide	1.72
Rhoa	Ras homolog gene family, member A	1.51
Icam2	Intercellular adhesion molecule 2	1.43
Csnk2b	Casein kinase 2, beta polypeptide	1.41
Llgl2	Lethal giant larvae homolog 2 (Drosophila)	1.31
Tjp2	Tight junction protein 2	1.20
Arhgef2	Rho/rac guanine nucleotide exchange factor (GEF) 2	1.19
Ilk	Integrin-linked kinase	1.02
Gsk3b	Glycogen synthase kinase 3 beta	-1.16
Tjp3	Tight junction protein 3	-1.18
Actn1	Actinin, alpha 1	-1.28
Rac1	Ras-related C3 botulinum toxin substrate 1	-1.48
Cdc42	Cell division cycle 42 (GTP binding protein)	-1.87
Mpdz	Multiple PDZ domain protein	-3.35
Pten	Phosphatase and tensin homolog	-3.79
Vapa	VAMP (vesicle-associated membrane protein)-associated protein A	-9.27

Supplemental Table 3: Tight junction gene expression of RMEPC on lung ECM with flow vs confluence on plastic. Green=upregulation on ECM, red=downregulation on ECM

Symbol	Entrez Gene Name	ECM flow vs plastic
MAGI2	membrane associated guanylate kinase, WW and PDZ domain containing 2	8.910
CDH5	cadherin 5, type 2 (vascular endothelium)	7.950
CLDN11	claudin 11	6.130
SPTB	spectrin, beta, erythrocytic	5.080
LLGL1	lethal giant larvae homolog 1 (Drosophila)	3.280
Tjp3	tight junction protein 3	1.800
CLDN18	claudin 18	1.720
CLDN15	claudin 15	1.530
CLDN19	claudin 19	1.100
TIAM1	T-cell lymphoma invasion and metastasis 1	-1.050
PARD6A	par-6 family cell polarity regulator alpha	-1.080
F11R	F11 receptor	-1.240
CLDN3	claudin 3	-1.310
CDC42	cell division cycle 42	-1.370
CSNK2B	casein kinase 2, beta polypeptide	-1.700
RHOA	ras homolog family member A	-1.880
PTEN	phosphatase and tensin homolog	-2.020
VAPA	VAMP (vesicle-associated membrane protein)-associated protein A, 33kDa	-2.060
CASK	calcium/calmodulin-dependent serine protein kinase (MAGUK family)	-2.170
AMOTL1	angiomotin like 1	-2.240
JAM3	junctional adhesion molecule 3	-2.390
CLDN12	claudin 12	-2.750
CSNK2A1	casein kinase 2, alpha 1 polypeptide	-2.770
RAC1	ras-related C3 botulinum toxin substrate 1 (rho family, small GTP binding protein Rac1)	-2.800
SPTAN1	spectrin, alpha, non-erythrocytic 1	-2.860
ICAM1	intercellular adhesion molecule 1	-2.980
CSNK2A2	casein kinase 2, alpha prime polypeptide	-3.060
Cdk4	cyclin-dependent kinase 4	-3.100
GSK3A	glycogen synthase kinase 3 alpha	-3.130
YBX3	Y box binding protein 3	-3.300
ILK	integrin-linked kinase	-3.320
SPTBN1	spectrin, beta, non-erythrocytic 1	-3.540
PARD6B	par-6 family cell polarity regulator beta	-3.640
SMURF1	SMAD specific E3 ubiquitin protein ligase 1	-3.840
MPDZ	multiple PDZ domain protein	-3.940

LLGL2	lethal giant larvae homolog 2 (Drosophila)	-3.990
CTNNB1	catenin (cadherin-associated protein), beta 1, 88kDa	-4.040
ARHGEF2	Rho/Rac guanine nucleotide exchange factor (GEF) 2	-4.050
ICAM2	intercellular adhesion molecule 2	-4.130
PRKCI	protein kinase C, iota	-4.410
GSK3B	glycogen synthase kinase 3 beta	-5.110
GNAI2	guanine nucleotide binding protein (G protein), alpha inhibiting activity polypeptide 2	-5.120
MAGI3	membrane associated guanylate kinase, WW and PDZ domain containing 3	-5.180
PARD3	par-3 family cell polarity regulator	-5.900
MPP5	membrane protein, palmitoylated 5 (MAGUK p55 subfamily member 5)	-6.200
TJP2	tight junction protein 2	-6.800
TJP1	tight junction protein 1	-6.810
ACTN4	actinin, alpha 4	-7.310
ACTN1	actinin, alpha 1	-7.460
ASH1L	ash1 (absent, small, or homeotic)-like (Drosophila)	-7.610
MARK2	MAP/microtubule affinity-regulating kinase 2	-8.300
CTTN	cortactin	-8.410
MLLT4	myeloid/lymphoid or mixed-lineage leukemia (trithorax homolog, Drosophila); translocated to, 4	-10.130
SPTBN2	spectrin, beta, non-erythrocytic 2	-10.890
SYMPK	symplekin	-10.910
TJAP1	tight junction associated protein 1 (peripheral)	-12.170
EPB41L1	erythrocyte membrane protein band 4.1-like 1	-18.050

Supplemental Table 4: Tight junction gene expression of RMEPC on lung ECM No-Flow vs Flow. Green=upregulation in No-Flow

Gene Symbol	Description	Fold Reg
Cdh5	Cadherin 5	27.09
Pard6a	Par-6 (partitioning defective 6,) homolog alpha (C. elegans)	18.56
Tjp3	Tight junction protein 3	9.09
Icam1	Intercellular adhesion molecule 1	7.59
Cldn18	Claudin 18	6.88
Tjp1	Tight junction protein 1	6.07
Prkci	Protein kinase C, iota	5.61
Icam2	Intercellular adhesion molecule 2	5.49
Tiam1	T-cell lymphoma invasion and metastasis 1	5.45
Tjap1	Tight junction associated protein 1	5.24
Csnk2a1	Casein kinase 2, alpha 1 polypeptide	5.06
Sptan1	Spectrin, alpha, non-erythrocytic 1	4.59
Jam3	Junctional adhesion molecule 3	3.30
Mpp5	Membrane protein, palmitoylated 5 (MAGUK p55 subfamily member 5)	3.09
Cldn15	Claudin 15	2.98
MLlt4	Myeloid/lymphoid or mixed-lineage leukemia (trithorax homolog, Drosophila); translocated to, 4	2.85
Sptbn1	Spectrin, beta, non-erythrocytic 1	2.85
Cdk4	Cyclin-dependent kinase 4	2.41
Amotl1	Angiomotin-like 1	2.40
Ash1l	Ash1 (absent, small, or homeotic)-like (Drosophila)	2.36
Cttn	Cortactin	2.34
Ybx3	Cold shock domain protein A	2.14
F11r	F11 receptor	2.02
Actn4	Actinin alpha 4	2.01
Ctnnb1	Catenin (cadherin associated protein), beta 1	1.86
Magi3	Membrane associated guanylate kinase, WW and PDZ domain containing 3	1.74
Mpdz	Multiple PDZ domain protein	1.70
Sptb	Spectrin, beta, erythrocytic	1.64
Sympk	Symplekin	1.61
Gsk3b	Glycogen synthase kinase 3 beta	1.56
Arhgef2	Rho/rac guanine nucleotide exchange factor (GEF) 2	1.44
Gnai2	Guanine nucleotide binding protein (G protein), alpha inhibiting 2	1.41
Rac1	Ras-related C3 botulinum toxin substrate 1	1.38
Pard3	Par-3 (partitioning defective 3) homolog (C. elegans)	1.37
Smurf1	Similar to RIKEN cDNA 4930431E10	1.34
Pard6b	Par-6 (partitioning defective 6) homolog beta (C. elegans)	1.29
Epb41l1	Erythrocyte membrane protein band 4.1-like 1	1.27
Actn1	Actinin, alpha 1	1.26

Rhoa	Ras homolog gene family, member A	1.26
Gsk3a	Glycogen synthase kinase 3 alpha	1.25
Csnk2a2	Casein kinase 2, alpha prime polypeptide	1.25
Cask	Calcium/calmodulin-dependent serine protein kinase (MAGUK family)	1.24
Csnk2b	Casein kinase 2, beta polypeptide	1.14
Pten	Phosphatase and tensin homolog	1.13
Tjp2	Tight junction protein 2	1.07
Ilk	Integrin-linked kinase	-1.01
Mark2	MAP/microtubule affinity-regulating kinase 2	-1.03
Cdc42	Cell division cycle 42 (GTP binding protein)	-1.16
Llgl2	Lethal giant larvae homolog 2 (Drosophila)	-1.26
Cldn3	Claudin 3	-1.27
Vapa	VAMP (vesicle-associated membrane protein)-associated protein A	-1.47
Cldn12	Claudin 12	-1.61

Supplemental Table 5: qPCR probes from Life Technologies (Grand Island, NY) used for additional gene expression experiments.

**Target**	**Assay/Probe ID**
Gapdh	Rn01775763_g1
Hif1a	Rn00577560_m1
Casp3	Rn00563902_m1
Vwf	Rn01492158_m1
Marco	Rn01408838_m1
Ldlr	Rn00598442_m1
Msr1 (Scara1)	Rn01488115_m1
Colec12 (Scara4)	Rn01531866_m1
Scarf1	Rn01470781_m1
Scarf2	Rn01404426_m1
Cd36 (Scarb3)	Rn00580728_m1
Itgav	Rn01485633_m1
Itgbl1	Rn01753534_m1
Itgb2	Rn01427948_m1
Itgb3	Rn00596601_m1
Itgb5	Rn01439348_m1
